# Treatment of a case of emphysematous pyelonephritis that presented with acute abdomen and pneumoperitoneum: a case report

**DOI:** 10.1186/s12882-015-0125-2

**Published:** 2015-08-01

**Authors:** Sang Hyun Park, Ki Hoon Kim

**Affiliations:** Department of Urology, Inje University College of Medicine, Haeundae Paik Hospital, Busan, 612-896 Republic of Korea; Department of Surgery, Inje University College of Medicine, Haeundae Paik Hospital, Busan, 612-896 Republic of Korea

**Keywords:** Emphysematous pyelonephritis, Pneumoperitoneum, Nephrectomy

## Abstract

**Background:**

Emphysematous pyelonephritis is a severe, life-threatening infection of the renal parenchyma and perinephric tissues. This condition is primarily encountered in patients with diabetes mellitus or ureteral obstruction, and is characterized by the production of intrarenal and perinephric gas. Emphysematous pyelonephritis is associated with a high degree of morbidity and a high mortality rate.

**Case presentation:**

A 72-year-old woman with a history of diabetes mellitus, hypertension, and renal calculi was referred to our emergency department following 6 days of abdominal pain. She suddenly developed pain in the entire abdomen, and was transferred. Physical examination was a distended abdomen with hypoactive bowel sounds. The tenderness was diffuse, but was most prominent in the right upper abdominal quadrant; moreover, rebound tenderness was noted. Laboratory tests revealed a white blood cell count of 4,480/mm^3^, platelet count of 17,000/mm^3^, creatinine level of 1.64 mg/dl, and serum glucose level of 603 mg/dl. Abdominal computed tomography indicated the presence of free air in the intraperitoneal cavity and right perirenal space, hydronephrosis of the right kidney, and stones in the right distal ureter. After 1 hour, the vital signs changed and she appeared to become drowsy. Therefore, the patient was transferred to the operation room for laparotomy. On exploration of the abdomen, 1.5 L of pus-colored fluid was removed. Although the abdominal viscera and pelvic organs were examined, hollow viscus perforation site could not be observed. Moreover, tissue necrosis and a perforation site were identified at the superior border of the right kidney. Thus, emphysematous pyelonephritis was diagnosed and she underwent right radical nephrectomy. After the surgery, the patient was admitted to the intensive care unit for postoperative management. Follow-up CT performed after 10 days showed fluid collection and hematoma at the nephrectomy site. Hence, percutaneous drainage was performed. Another follow-up computed tomography after 3 weeks indicated that the fluid collection at the nephrectomy site had nearly disappeared.

**Conclusions:**

We believe that cases with free intraperitoneal air should promptly undergo laparotomy to identify the cause of the pneumoperitoneum. Moreover, an immediate nephrectomy may be effective for the treatment of emphysematous pyelonephritis in cases with poor prognostic factors.

## Background

Emphysematous pyelonephritis is a severe, life-threatening infection of the renal parenchyma and perinephric tissues. This condition is primarily encountered in patients with diabetes mellitus (DM) or ureteral obstruction, and is characterized by the production of intrarenal and perinephric gas [1, 2]. Emphysematous pyelonephritis is associated with a high degree of morbidity and a high mortality rate. In the present report, we describe a case of emphysematous pyelonephritis that presented with pneumoperitoneum.

## Case presentation

**Fig. 1:**
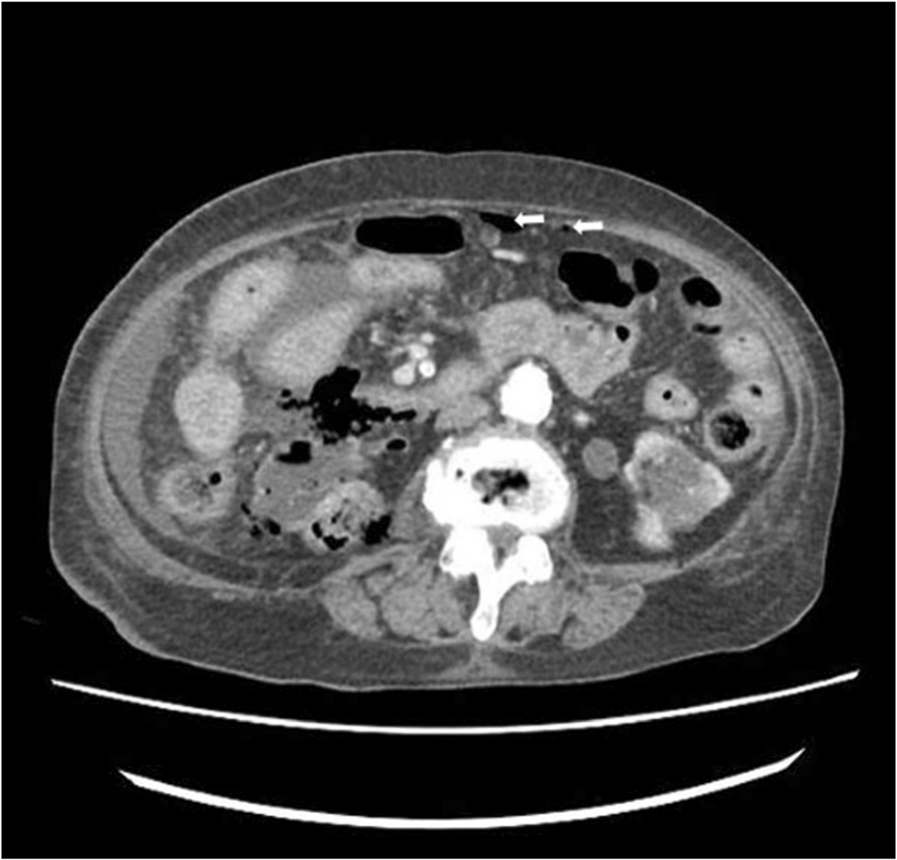
Abdominal CT finding. Axial view showing gas in the right renal parenchyme and perirenal space and intraperitoneal air (arrows)

**Fig. 2:**
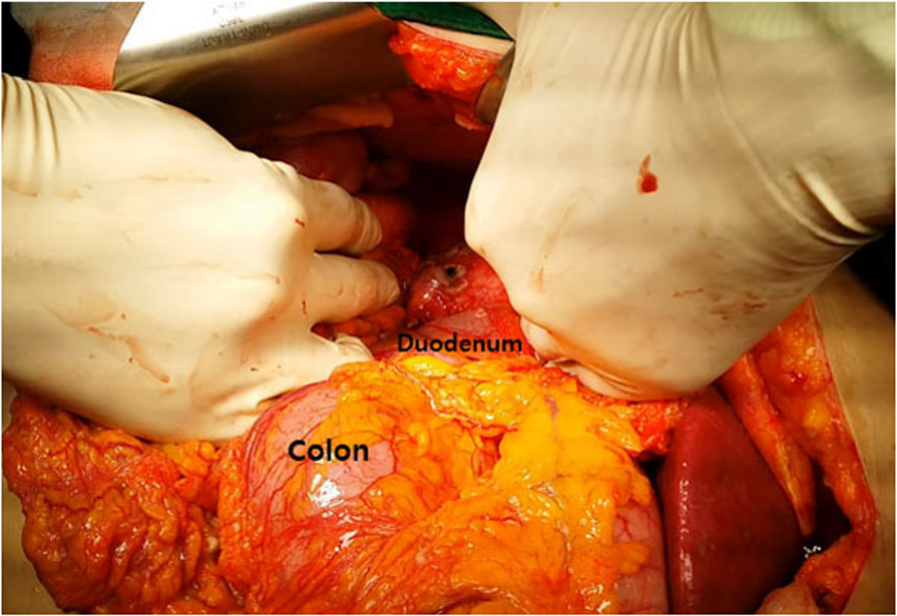
Intraoperative finding. A perforation site was identified at the superiolateral border of second portion of duodenum

A 72-year-old woman with a history of DM, hypertension, and renal calculi was referred to our emergency department following 6 days of abdominal pain. She was initially started on intravenous ciprofloxacin at another hospital; despite this treatment, her symptoms persisted and gradually exacerbated. She suddenly developed pain in the entire abdomen, and was therefore transferred to our hospital for further management. Her initial vital signs indicated a blood pressure of 140/90 mmHg, heart rate of 120 beats per minute, temperature of 37.9 °C, and respiratory rate of 20 breaths per minute. Physical examination on admission was a distended abdomen with hypoactive bowel sounds. The tenderness was diffuse, but was most prominent in the right upper abdominal quadrant; moreover, rebound tenderness was noted. Laboratory tests revealed a white blood cell count of 4,480/mm^3^ with 86 % granulocytes, hemoglobin level of 11.2 g/dl, platelet count of 17,000/mm^3^, creatinine level of 1.64 mg/dl, blood urea nitrogen level of 44.4 mg/dl, and serum glucose level of 603 mg/dl. Abdominal computed tomography (CT) indicated the presence of free air in the intraperitoneal cavity and right perirenal space, hydronephrosis of the right kidney, fluid and fat infiltration in the right perirenal space, and stones in the right distal ureter (Fig. 1). Hence, the patient was started on intravenous piperacillin-tazobactam plus ciprofloxacin, as well as fluid resuscitation on arrival. After 1 hour, the vital signs changed—the blood pressure decreased to 90/50 mmHg and temperature increased to 38.6 °C. Furthermore, she appeared to become drowsy. Therefore, the patient was immediately transferred to the operation room for laparotomy. On exploration of the abdomen, 1.5 L of pus-colored fluid was removed. Although the abdominal viscera and pelvic organs were examined, hollow viscus perforation site could not be observed. Moreover, tissue necrosis and a perforation site were identified at the superior border of the right kidney (Fig. 2). Thus, emphysematous pyelonephritis was diagnosed and she underwent right radical nephrectomy. During the surgery, the patient’s vital signs were unstable, and hence, vasopressin was intravenously administered. After the surgery, the patient was admitted to the intensive care unit for postoperative management. Norepinephrine was administered to maintain the blood pressure, and was tapered on postoperative day 2. The patient was weaned from ventilator support and extubated on postoperative day 4. Follow-up CT performed 10 days after the initial study showed fluid collection and hematoma at the nephrectomy site. Hence, percutaneous drainage was performed. Another follow-up CT after 3 weeks indicated that the fluid collection at the nephrectomy site had nearly disappeared. The patient was discharged to home in a stable condition, and was advised to attend follow-up visits at the surgery and nephrology departments.

## Discussion

Emphysematous pyelonephritis—a serious complication of upper urinary infection—has been defined as an acute, severe, necrotizing infection of the renal parenchyma and perirenal tissue, which results in the development of gas within the renal parenchyma, collecting system, or perinephric tissue [1–3]. It is usually associated with DM and/or urinary obstruction such as stones [4]. However, the mechanism of gas formation and pathogenesis of emphysematous pyelonephritis are unclear. Huang and Tseng [5] hypothesized that the high level of blood glucose in patients with DM may provide gas-forming microorganisms with a more favorable environment for gas formation via mixed acid fermentation of glucose. However, bacterial gas production does not fully explain the pathologic and clinical manifestations of this condition.

Emphysematous pyelonephritis is diagnosed by the identification of gas in renal or perinephric tissue [6]. CT is reportedly the most sensitive method for detecting gas in and adjacent to the kidney [1, 7–9]. Based on CT findings, Wan et al [10] proposed that emphysematous pyelonephritis can be classified into two types, in order to determine the prognosis and guide therapy. Furthermore, Huang and Tseng [5] classified emphysematous pyelonephritis into four types based on the radiological findings on CT. Thus, the prognosis and treatment course of cases with emphysematous pyelonephritis can be determined according to classifications based on CT findings. However, pneumoperitoneum caused by emphysematous pyelonephritis has not been included in any of the classification systems based on CT findings.

Emphysematous pyelonephritis can be seen with pneumoretroperitoneum due to its location within the retroperitoneum, and the perirenal fascial boundaries may be disrupt in fulminant infection, allowing the air migrate into other compartments. Cases of pneumoperitoneum caused by emphysematous pyelonephritis are rare. To our knowledge, only two other reports in the literature have described cases of pneumoperitoneum with emphysematous pyelonephritis [11, 12]. Therefore, a standard treatment method has not yet been established. In both the cases reported in the literature, laparotomy was promptly performed. Moreover, drainage and delayed nephrectomy were performed in one case whereas only drainage was performed in the other case. In the present case laparotomy and radical nephrectomy were promptly performed. This surgical treatment was decided because the patient complained of whole abdominal tenderness and rebound tenderness, pneumoperitoneum was noted on CT, her condition deteriorated, and she had severe risk factors. Falagas et al [13] reported that the significant risk factors for mortality in such cases include conservative treatment alone, bilateral emphysematous pyelonephritis, type I emphysematous nephritis, and thrombocytopenia. The presence of a systolic blood pressure of <90 mmHg, serum creatinine level of >2.5 mg/dL, and disturbance of consciousness are also reportedly associated with increased mortality in these cases. Huang and Tseng suggest that cases of extensive emphysematous pyelonephritis with a fulminant course (≥2 risk factors) should promptly undergo nephrectomy in order to achieve the best outcome.

In addition, the presence of pneumoperitoneum usually indicates visceral perforation. Prompt surgical intervention is usually required in these patients to reduce the degree and magnitude of enteric contamination within the peritoneal cavity. Thus, surgery is the main treatment for cases of emphysematous pyelonephritis and pneumoperitoneum.

## Conclusions

We believe that cases with free intraperitoneal air should promptly undergo laparotomy to identify the cause of the pneumoperitoneum. Moreover, an immediate nephrectomy may be effective for the treatment of emphysematous pyelonephritis in cases with poor prognostic factors.

## Consent

Written consent was obtained from the patient for this publication. A copy of written consent is available for review by the editor of this journal.

## Abbreviations

DM: Diabetes mellitus
CT: Computed tomography
